# Further Researches on Ethylate of Sodium in the Treatment of Nævus and Other Forms of Disease

**Published:** 1881-05

**Authors:** Benjamin Ward Richardson


### FURTHER RE EARCHES ON ETHYLATE OF SODIUM IN THE TREATMENT OF NÆVUS AND OTHER FORMS OF DISEASE.

BY BENJAMIN WARD RICHARDSON, M. D., F. R. S.

THE introduction of sodium ethylate, like that of amyl nitrite, into the practice of medicine and surgery was due to the researches on the amyls, ethyls, and alcohols, which I had the honor to carry out for my reports to the British Association for the Advancement of Science between the years 1863 and 1871. In observing the action of the more ordinary form of alcohol—the ethylic—on the blood and the tissues, it occurred to me to inquire what difference of action would occur if an alcohol was used in which sodium or potassium takes the place of the remaining atom of hydrogen which belongs to the water molecule present in the alcohols—that is to say, what would be the action of ethylic alcohol if the radical ethyl which replaces one of the hydrogens of the water was united with a metal-like sodium or potassium, instead of hydrogen? The ethylates of sodium and potassium, sodium and potassium alcohols, were at that time well known as chemical substances; but they had not been used as medicinal agents, nor had their employment as remedies been suggested. It struck me however, that they might come in as serviceable caustics, and at the same time as antiseptics.

In the treatment of vascular nævus (cutaneous) I have had since my last report nine cases, each of which has done well. They have all been in children, and some of them have been cases in which other modes of treatment had, previously, been tried. It may be well to give a brief review of these cases in order.

The first was an instance of the common form of nævus on the scalp in an infant three months old. After perfect recovery from vaccination, the treatment commenced in the usual way by the application of ethylate over the growth, by means of the glass-rod. The nævus was small, not larger than a fair-sized hazel nut. The first application caused a dense scale to form, which was loose and removable on the fifth day. The ethylate was then re-applied, and five days later, when the new scale was removed, the nævus was reduced to the size of a small bean. It remained in this state during three further applications of the ethylate, being much longer under treatment than I had expected after the second application. On the seventh application it was nearly removed, and one additional touch a fortnight later completely removed it. No constitutional symptoms interfered with the course of the treatment, and no scar remained.

In the next example the treatment was almost identical both in respect to mode and to result; but as the patient was very restive and screamed extremely when the ethylate was applied, advantage was taken to make the application when the infant was in deep sleep. The plan succeeded so well that I ventured to suggest its general adoption in young children whenever the nævus is in a situation where it can be easily got at, and whenever an intelligent nurse or parent can be taught to make use of the solution in a safe and efficient manner. In the case in question the nævus was quite removed in the course of six weeks, and it can scarcely be said that any pain at all was inflicted. No scar has been left.

The third illustration was not so favorable as the two above named, although in the end the patient did well. The nævus, again on the scalp, was near to the anterior fontanelle, the fontanelle itself being large. A cerebral pulsation, distinctly marked, extended to the nævus, itself. I judged at first that there was a communication from the external nævus to another vascular enlargement within the cranial arch, but this turned out to be incorrect. The child was eight months old, was pale and feeble, with a large head. The nævus originally was of the size of a small walnut, and very prominent. The first application of the ethylate gave rise to a large and firm scale of the usual kind, which was removable on the fifth day. The second application caused a great deal of pain, and the crust which formed remained for a week immovable. When the crust was at last loosened and lifted up, the nævus was found to be reduced in size, but excessively red and vascular. It was freely treated with the ethylate, and again a dense and firm crust resulted. Four days after this the crust was quite firm, and on the eighth day, as the crust was still immovable by the ordinary method of lifting it up with forceps, I, forgetful for the moment of my own instructions, directed the nurse to apply a poultice and bring the patient to me again when the crust was softened. Two days later the child was brought very unwell. It was feverish; temperature 103°. There was an odor of decomposition from nævus, and a dark sphacelus. I immediately dried the part most carefully with absorbent wool, and applied ethylate freely, covering with dry wool lightly. The good effect was immediate; the decomposition was arrested, a new and firm crust was formed, and from that time the recovery went on favorably, the nævus being destroyed, and scarcely a scar being left. The error I made in this instance consisted in the use of moisture from the poultice. It was this which set up the decomposition, and it was the decomposition and the secondary absorption which set up the febrile condition. I name the fact, and the temporary blunder involved in it, in order to re-inforce the point of practice that in using the ethylate water must never, under any pretense, be introduced as an adjunct. The mention of that example leads me to name the further practical suggestion that the removal of the crust, or the attempt to remove it too quickly, is not a good plan. It is best to let the crust come away of itself, or, at all events, to become so loose that it can easily be lifted away. We may take it as a general rule that so long as the crust is firm, the nævus is contracting. Now, therefore, instead of removing the crust entirely, I am content to take off those parts only which are loose, and then to apply to another touch of ethylate over the parts that have been removed. Certainly, all violence in removal is essentially mischievous. It causes pain; it often causes bleeding; and it prolongs, instead of expediting, recovery. When the crust is depressed in the centre it may be pierced with a needle, and little ethylate may be introduced through the openings. This plan answers exceedingly well.

The next four succeeding instances of nævus treated with the ethylate presented no special features, except that in one of them there were two growths on the same patient. The question here was raised, whether it was best to treat both the tumors at the same, or one at a time. The last-named course was adopted, and with very good results. The nævus first subjected to treatment was removed in twenty-four days, and after a week the other one, which was smaller, was removed in a little less than the same period of time. The four cases did well, and in one only is there any appreciable scar. The patients were all young. The youngest was six months; the eldest a little under two years of age.

The employment of sodium ethylate in the treatment of nasal polypus led me to think of using it in ozœna. Here I have had an experience in a long-standing but not extremely severe example of the disease. In this instance, in a young girl, the affection was confined to one nostril, from which there was constant fetid discharge, with evidence of ulceration of the mucous membrane at the distance of about an inch within the cavity. In this case I diluted the ethylate with absolute alcohol to one-half, and then introduced a pellet of cotton wool, by means of the curved forceps, into the canal, up to the ulcerated surface. Withdrawing the forceps I left the wool in the cavity for five minutes, and then withdrew it. The application was attended with severe pain, and was followed by an irritation which lasted for three or four days; but the secretion was so much arrested and the offensive odor so greatly relieved that the patient willingly permitted a repetition of the treatment for three successive times. The discharge thereupon entirely ceased, and the odor has not again been noticeable after a period of several months, so that I hope a cure has been effected.

In these cases, again, the theory of the action of the ethylate as a means of cure is quite in accord with the promise suggested by theory, and that, too, though the bony structure should be the seat of ulceration. For I find that the action of the ethylate on soft bone and cartilage is to destroy texture by the decomposition of water, and to fix or pectise the gelatinous matter. The ethylate may thus again be extended in application on this basis of operation. It may, I think, be often successfully used for the destruction of caries or necrosed bone, attended with purulent discharge.—*London Lancet.*

